# American Dental Association

**Published:** 1883-09

**Authors:** 


### AMERICAN DENTAL ASSOCIATION.

### FIRST DAY.

The twenty-third annual meeting of this Society was held in the parlors of the International Hotel, at Niagara, commencing Aug. 7th, 1883.

The President, Dr. W. H. Goddard, having died during the year, the First Vice President, Dr. Geo. J. Friedrichs, of New Orleans, presided, and opened the meeting with the customary address.

The President's chair stood unoccupied and draped in mourning, and just behind it hung a picture of the lamented Dr. Goddard, also appropriately draped.

The Executive Committee in its recommendations set apart the time from nine A. M. till two P M., and from eight P. M. till adjournment for the Association work, leaving the afternoon from two till eight for work in the sections, clinics, etc., etc., the evening session for the first day being omitted. After some discussion the recommendation was adopted.

Dr. Pierce moved to appoint a committee to draft resolutions in relation to the death of Dr. Marshall H. Webb.

Dr. Odell moved a similar resolution in relation to the death of Dr. Wm. H. Allen.

Dr. Rehwinkel moved a similar resolution regarding the death of our late President, Dr. Wm. H. Goddard.

The Publication Committee reported that the proceedings had been printed by the S. S. White Manufacturing Co. without expense to the Society, they having been given the exclusive right to the publication of all papers read.

SECOND DAY.

The meeting was called to order by the acting President, Dr. Friedrichs.

Dr. Pierce, Chairman of Committee on Resolutions concerning the death of Dr. Webb, reported, and was followed by some eulogistic remarks in relation to the life of Dr. Webb, by Dr. J. Taft.

Section V was called upon but not being ready, Section VI—Pathology, Therapeutics and Maderia Medica—was called, and response made by the Secretary, Dr. Dudley, who announced two papers as ready for presentation, one on "Syphilitic Teeth," by Dr. G. A. Friedrichs, of New Orleans, and one by Dr. A. W. Harlan, of Chicago, on "Treatment of Pyorrhœa Alveolaris."

The first mentioned paper was then read and discussed at considerable length.

Dr. Abbott said the distinctive appearance presented by syphilitic teeth is as though they had been turned upside down, having square crowns, or rather with the end smaller than the neck.

Dr. Barrett had watched to find the "creases" that have been charged upon syphilis, but is satisfied that there is no such thing as a true, unvarying syphilitic type of teeth.

Dr. Darby spoke of some models which he has made (he regretted that they were not at hand to exhibit) from the teeth of people in the lower walks of life, which he considered to be a strong point in favor of the truthfulness of the record, wherein the distinctive character of the teeth might be studied and the origin of the abnormality in syphilis authenticated.

Dr. Pierce said the paper presented us the facts as we have them in literature to-day. Observation has taught dentists that other causes produce these appearances upon the teeth.

Dr. Atkinson here put in a plea for the study of Embryology and Histology.

Dr. Harlan's paper on "Treatment of Pyorrhœa Alveolaris" was then read, giving the author's experience during the past ten months in the treatment of many cases of this disease, and claiming a complete success in nearly every instance. He first thoroughly removes the deposits with instruments, then with cotton wrapped upon the end of an appropriate broach he rubs the exposed roots and necks of the teeth thoroughly, the cotton being from time to time dipped into a solution of iodo-chloride of zinc, after which he injects into each pocket dioxide of hydrogen ($H.^2$ $O.^2$), this being a most powerful germicide, and a diagnostic agent of the first importance in detecting the presence of pus. The pockets being first carefully dried with absorbing cotton, any pus remaining or exuding will cause the $H.^2$ $O.^2$ to effervesce, so that the eye can readily perceive it.

Treatment at intervals of four days will usually effect a radical cure in from three to four applications.

*Dr. Bodecker.*—I have been studying this subject for years. It is not certain that bacteriæ produce inflammation; they are always present as the result, not necessarily as the cause. Antiseptics are, of course, needed, but only in their mildest form.

The preparation of zinc recommended, although valuable, if too freely exhibited, will produce injury. I use first the iodo-chloride of zinc, following with a solution of tartrate of chinoline of the strength of one dram to two oz. of water. Have seen results under this treatment that were simply marvelous.

*Dr. Darby* uses iodoform for this purpose, and also finds the same application of great service in the treatment of odontalgia, although its odor is a serious objection to its use. As another remedy of value employs saltpetre, or ordinary nitre. Has had the best of results by its use.

*Dr. Abbott.*—The best remedy I have ever used in this disease is properly handled instruments. I find little necessity for anything further.

*Dr. Atkinson.*—I see always a disposition to push the inquiry to the ultimate. Are the hard tissues well understood?

Inflammation is never anything but an arrest of nutrient activity. There is always a great disposition on the part of very sharp minds to say: "I do not believe it." If we fulfilled all the demands of the body we would have no disease at all. Statistics are all well enough if faithfully made, but require proper interpretation. We need not say, "we hope to get new process;" we *can* get it!

*Dr. Abbott.*—Am sorry that our friend Atkinson did not follow back the changes of tissue to its embryonal conditions, in rebuilding from which you will get the reproduction of alveolar process.

After some further remarks by members the subject was passed.

Dr. Bodecker, of New York, read a paper on "The Action of Arsenious Acid upon Dental Pulp Tissues." He requested that discussion upon the subject be deferred until members had had an opportunity to examine with the microscope some specimens which he had brought for the purpose of illustrating points in the paper.

An abstract of Dr. Bodecker's paper will be found upon page 468 of this number.—EDITOR.

Dr. Richardson, of London, Eng., was introduced to the Association, and granted the privileges of the floor.

Dr. Odell, Chairman of Committee on Resolutions in relation to the death of Dr. Wm. H. Allen, reported appropriate resolutions and followed their reading by an historical sketch of the deceased. It was voted that a memorial page be inserted in the transactions.

Dr. Rehwinkel, Chairman of the Committee on Resolutions in relation to the death of Dr. Wm. H. Goddard, also reported suitable resolutions.

Dr. N. W. Kingsley was then, by resolution, requested to address the Association upon the subject of "Articulate Speech," which he proceeded to do, illustrating his remarks by means of the blackboard.

THIRD DAY.

After the transaction of the general routine business, Dr. Pierce, Chairman of the Committee on Prize Essays, reported as follows:

"Your committee to whom was assigned the duty of deciding upon the merits of essays upon 'The Etiology of Dental Caries,' offered for a prize of two hundred dollars, which was last year appropriated by this Association for the purpose, would respectfully report that but one essay has been received, and that from the hands of Dr. W. D. Miller, of Berlin, Germany.

The committee have carefully read this, and while the views contained therein are not all original, many of his experiments, which are in detail and made for the purpose of confirming his theory, have not been previously published. Your committee, would, therefore, in view of the original work which the author has prosecuted the last two years, the results of which are given in this paper, award to the essayist the two hundred dollars appropriated for the purpose." The report was adopted.

Section VII—Physiology and Etiology—was then called and the Chairman read the report, but announced that there were no papers belonging to the Section to be read.

Section I being called—Artificial Dentistry, Chemistry and Metallurgy—the Chairman, Dr. T. L. Buckingham, read the report. He announced that the Section had no papers to present, but opened the discussion by some remarks upon chemistry.

Dr. E. Parmly Brown presented some new crowns for natural roots, consisting of a plate of platinum with enameling to represent the visible portion, the interior to be filled with amalgam or any other desired material with which to make the attachment.

Section II—Dental Education—was called and the report was, in the absence of the Chairman, Dr. L. D. Shephard, read by Dr. Pierce. He said that one paper had been presented, but on account of its general character it was not recommended to the Association. He presented also the following resolution, which was amended and finally adopted as follows:

*Resolved*, that the interests of the profession and advanced dental education both demand that all dental educational institutions shall require that every student, before being admitted to examination for the degree of Doctor of Dental Surgery, shall have taken two full courses of lectures.

Dr. Pierce also offered the following, which was passed:

*Resolved*, That the American Dental Association deems it adverse to the interests of the dental profession for any State Board of Examiners to confer a title or degree of any nature.

Dr. Buckingham thought that a professor in a Dental College should not be a member of any State Board.

Dr. Rehwinkel explained the difference between the diploma of a college and the certificate of a State Board of Censors.

Dr. Pierce believed that the time would come when the State Board could be abandoned.

Dr. Allport thought that as the standard of dental education was raised there would be an increased necessity for dental examining boards.

Dr. Hayhurst spoke strongly in favor of the colleges, and reminded members that a State Board certificate was mere waste paper outside the State where it was granted.

Dr. Odell desired that the paper upon education be read.

Dr. Crouse objected.

Section III was called—Dental Literature and Nomenclature—and the report was read by Dr. J. Taft, Chairman. Two papers were presented, one by Dr. Atkinson, and the other by Dr. G. J. Friedrichs, both of which were read. The Chairman said that he had mislaid a paper by Dr. Francis, but he would forward it in time for the general report.

Dr. Crouse said that there were not more than fifteen thousand dentists in the United States, and he was in favor of weeding out most of the dental journals ; that one journal well boiled down, and containing a condensed mass of literature, was all that was needed.

Dr. Abbott said that nearly all dentists were now taking dental journals, and that the call for a literature was increasing. There can be no liberal profession without a literature, and it would be a black day for dentistry when the views of the last speaker prevailed, for it would carry us back into the dark ages, the time of usurpations, and monopolies and inquisitions. In this day intelligence is free, and it is mainly disseminated through the means of a periodical literature.

At the evening session the regular election was held, which resulted in the choice of Saratoga as the next place of meeting, and the following list of officers :

President—Dr. E. T. Darby, Philadelphia, Pa.

1st Vice President—Dr. C. S. Stockton, Newark, N. J.

2nd Vice President—Dr. T. T. Moore, Columbia, S. C.

Cor. Secretary—Dr. A. W. Harlan, Chicago, Ill.

Rec. Secretary—Dr. Geo. H. Cushing, Chicago, Ill.

Treasurer—Dr. Geo. W. Keeley, Oxford, Ohio.

Executive Committee—Dr. G. J. Friedrichs, New Orleans, La. ; Dr. S. G. Perry, New York, N. Y. ; Dr. W. N. Morrison, St. Louis, Mo.

The President appointed as the local committee of arrangments :

Dr. M. L. Rhein, of New York.

Dr. F. M. Odell, of New York.

Dr. C. F. Rich, of Saratoga.

FOURTH DAY.

After the officers elect were installed, the salary of the Secretary was increased to $200 per annum.

Section IV—Operative Dentistry—being called, the report was read by Dr. E. T. Darby, the Chairman. The discussion was extended, but somewhat desultory.

After the reception of the final reports the customary votes of thanks were extended to all to whom the Association was indebted for courtesies.

The regular order of business was then suspended, and the resolution awarding the prize for the essay upon Etiology was reconsidered, and referred back to the committee for another year.

After reading the minutes of the morning proceedings, the Society adjourned for one year.

---

